# Inherited selective cobalamin malabsorption in Komondor dogs associated with a *CUBN* splice site variant

**DOI:** 10.1186/s12917-018-1752-1

**Published:** 2018-12-27

**Authors:** John C. Fyfe, Shelby L. Hemker, Alycia Frampton, Karthik Raj, Peter L. Nagy, Kristi J. Gibbon, Urs Giger

**Affiliations:** 1 Laboratory of Comparative Medical Genetics, Michigan State University, 567 Wilson Road, East Lansing, MI 48824 USA; 2 Department of Microbiology & Molecular Genetics, Michigan State University, 567 Wilson Road, East Lansing, MI 48824 USA; 3 Section of Medical Genetics, University of Pennsylvania, 3900 Delancey Street, Philadelphia, PA 19104-6010 USA; 4 Laboratory of Personalized Genomic Medicine, Department of Pathology & Cell Biology, Columbia University - College of Physicians & Surgeons, 630 West 168th Street, New York, NY 10032 USA; 5 Oregon Veterinary Referral Associates, 444 B Street, Springfield, OR 97477 USA; 6 Present address: Department of Pediatrics, Division of Nephrology, UPMC Children’s Hospital of Pittsburgh, University of Pittsburgh, 4401 Penn Ave, Pittsburgh, PA 15224 USA; 7 Present address: MNG Laboratories™, 5424 Glenridge Drive NE, Atlanta, GA 30342 USA; 8 Cottonwood Heights, USA

**Keywords:** Vitamin B_12_, Amnionless, Cubilin, Inborn error of metabolism, Methylmalonic aciduria, Animal model, Failure to thrive

## Abstract

**Background:**

Three Komondor dogs in a small family and 3 sporadic cases exhibited a constellation of signs that included juvenile-onset of failure-to-thrive, inappetence, vomiting and/or diarrhea, and weakness. In each we documented dyshematopoiesis, increased anion gap, methylmalonic acidemia/-uria, and serum cobalamin deficiency. Urine protein electrophoresis demonstrated excretion of cubam ligands. All clinical signs and metabolic abnormalities, except proteinuria, were reversed by regular parenteral cobalamin administration. The pattern of occurrence and findings in the disorder suggested an autosomal recessive inheritance of cobalamin malabsorption with proteinuria, a condition in humans called Imerslund-Gräsbeck syndrome. The purpose of this study was to determine the molecular cause of this disorder in Komondors.

**Results:**

Whole genome sequencing of two affected Komondor dogs of unknown relatedness and one parent and a clinically-normal littermate of an affected dog revealed a pathogenic single-base change in the *CUBN* intron 55 splice donor consensus sequence (NM_001003148.1: c.8746 + 1G > A) that was homozygous in affected dogs and heterozygous in the unaffected parents. Alleles of the variant co-segregated with alleles of the disease locus in the entire family and all more distantly-related sporadic cases. A population study using a simple allele-specific DNA test indicated mutant allele frequencies of 8.3 and 4.5% among North American and Hungarian Komondors, respectively.

**Conclusions:**

DNA testing can be used diagnostically in Komondors when clinical signs are suggestive of cobalamin deficiency or to inform Komondor breeders prospectively and prevent occurrence of future affected dogs. This represents the third cubilin variant causing inherited selective cobalamin malabsorption in a large animal ortholog of human Imerslund-Gräsbeck syndrome.

**Electronic supplementary material:**

The online version of this article (10.1186/s12917-018-1752-1) contains supplementary material, which is available to authorized users.

## Background

Cobalamin (vitamin B_12_) is an essential micronutrient for mammals that, when metabolized to the forms 5′-adenosyl cobalamin and methyl-cobalamin, serves as a cofactor for two enzymes, methylmalonyl-CoA mutase and methionine synthase, respectively [1]. Deficiency of these cofactors at the cellular level inhibits the respective enzymatic activities. Unprocessed substrates are observed as methylmalonic acidemia/-uria and homocysteinemia, while secondary metabolites disrupt ammonia elimination, glucose homeostasis, and nucleotide synthesis. Thus, clinical signs of severe cobalamin deficiency and its metabolic effects are far reaching and include dyshematopoiesis, gastrointestinal (GI) disturbances, post-natal developmental delay, and life-threatening metabolic derangements [2].

Cobalamin is synthesized only by certain microorganisms, and monogastric species obtain this vitamin from animal-derived foods via a complex receptor-mediated mechanism of the GI tract initially delineated in William Castle’s investigations of pernicious anemia [3]. Successful cobalamin absorption is a sequence of protein-binding events that each depend on the longitudinal secretory and absorptive organization of the GI tract [4]. The daily dietary cobalamin requirement in dogs is 2–3 μg, and there is a large flux of enterohepatic recirculation of the vitamin. Cobalamin deficiency is most often caused by GI malabsorption, rather than dietary deficiency, occurring either as generalized GI malabsorption or as a selective process in which cobalamin is the only dietary component that is lost. Hereditary selective cobalamin malabsorption is caused mainly by defects that interrupt secretion or function of intrinsic factor (IF), a protein product of gastric parietal cells in humans and pancreatic duct cells in dogs, or of cubam, the highly specific, IF-cobalamin receptor on the apical, brush-border membrane of epithelial cells in the distal small intestine [5]. In the ileum, cubam selectively mediates absorption of the IF-cobalamin complex from food, and absorbed cobalamin binds transcobalamin, a plasma transport protein for delivery of the vitamin to cells [4].

In addition to the distal small intestine, cubam is expressed in renal proximal tubular epithelial cells. In the kidney, cubam mediates reabsorption of a variety of protein ligands, such as albumin, apo AI (involved in lipid metabolism), haptoglobin, and vitamin D-binding protein from the glomerular filtrate [6–9]. Therefore, cubam dysfunction not only causes intestinal cobalamin malabsorption but also a mild proteinuria of mid- to low-molecular weight proteins that are cubam ligands. The ligand-specific proteinuria is clinically benign but is a useful diagnostic marker of cubam dysfunction.

The functional cubam complex is composed of cubilin (CUBN) and amnionless (AMN) subunits [10]. The recently solved crystal structure [11] of cubam confirms that three molecules of CUBN form an extracellular ligand-binding trimer [12]. A single transmembrane AMN molecule provides apical membrane anchorage of the trimer and cytoplasmic signaling that initiates clathrin-mediated endocytosis [13]. In humans [14, 15] and dogs [16–20] various gene variants in both *CUBN* and *AMN* are found causing hereditary selective intestinal cobalamin malabsorption with mid- to low-molecular weight proteinuria, an autosomal recessive trait also known as Imerslund-Gräsbeck syndrome (I-GS) [21, 22]. Though genetically heterogeneous, the disorder in dogs is phenotypically quite similar, and the various causative *AMN* or *CUBN* variants are breed-specific. Description of cobalamin malabsorption in dogs caused by *AMN* variants (OMIA 000565–9615) and *CUBN* variants (OMIA 001786–9615) is curated in the Online Mendelian Inheritance in Animals data base (https://omia.org).

We describe here autosomal recessive selective intestinal cobalamin malabsorption with proteinuria in Komondor dogs characterized by failure to thrive, dyshematopoiesis, and metabolic disturbances during the juvenile period, as occur in I-GS of other dog breeds and human patients. The purpose of this study was to determine the molecular defect underlying I-GS in Komondors and to determine the disease-allele frequency in Komondor populations of Hungary and North America.

## Results

### Clinical signs of I-GS in Komondors

Owners of affected Komondors first noted signs of failure-to-thrive between 2.5 and 5 months of age that were progressive until institution of parenteral cobalamin administration. Typical clinical signs included inappetence, vomiting, diarrhea, failure to thrive, and weakness. One dog exhibited a fine head tremor, and another had intermittent seizures. On physical examinations the puppies were lethargic and underweight but of near-normal linear size.

**Fig. 1:**
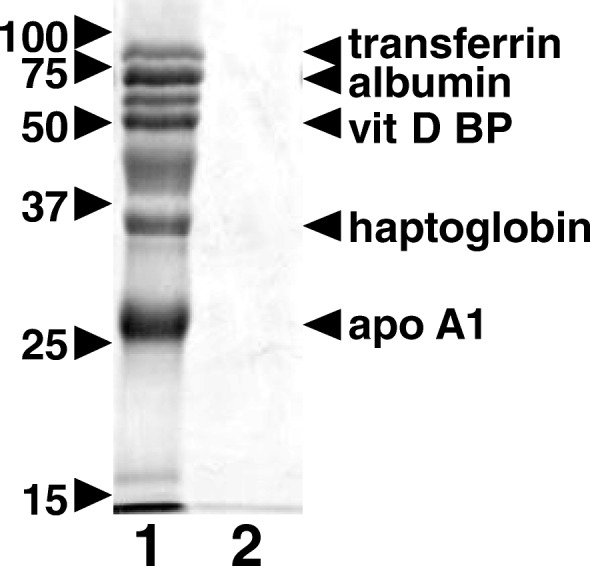
Urine protein analysis of Komondor littermates. Urine samples containing 200 μg creatinine were desalted and concentrated by centrifugation through a 10 kDa molecular weight cutoff membrane. Proteins were separated by 15% SDS-PAGE and visualized by silver staining. Lane 1 shows urine proteins obtained from an affected dog (case 5, Additional file 1) after cobalamin replenishment and during metabolic remission. Lane 2 shows urine proteins from a heterozygous but clinically healthy littermate of case 5

Routine complete blood counts revealed a non-regenerative, normocytic, normochromic anemia or low-normal hematocrit. Neutrophil counts were between 900 and 3700/μL (normal range for puppies 5000-12,000/μL) and some appeared toxic and/or hypersegmented. The dogs also had a mild thrombocytopenia. Serum chemistry indicated mildly increased blood urea nitrogen, normal serum creatinine concentrations, and increased anion gaps, suggesting a metabolic acidosis. Serum bile acid concentrations were normal. Serum cobalamin concentrations were undetectable (<150 ng/L) or below the lower limited of normal (250 ng/L). Urinalysis showed mild to moderate proteinuria by dipstick (2+ to 4+ in concentrated urine). Urine metabolic screens showed strongly positive methylmalonic acid (MMA) spot test results, and quantitative organic acid analyses revealed extremely high MMA concentrations of 20,000–37,000 mmol/mol creatinine (normal upper limit = 2 mmol/mol creatinine). The mid- to low-molecular weight proteins found in urine from affected dogs were identified as cubam ligands, including transferrin, albumin, vitamin D binding-protein, haptoglobin, and apo A1 (Fig. 1).

**Fig. 2:**
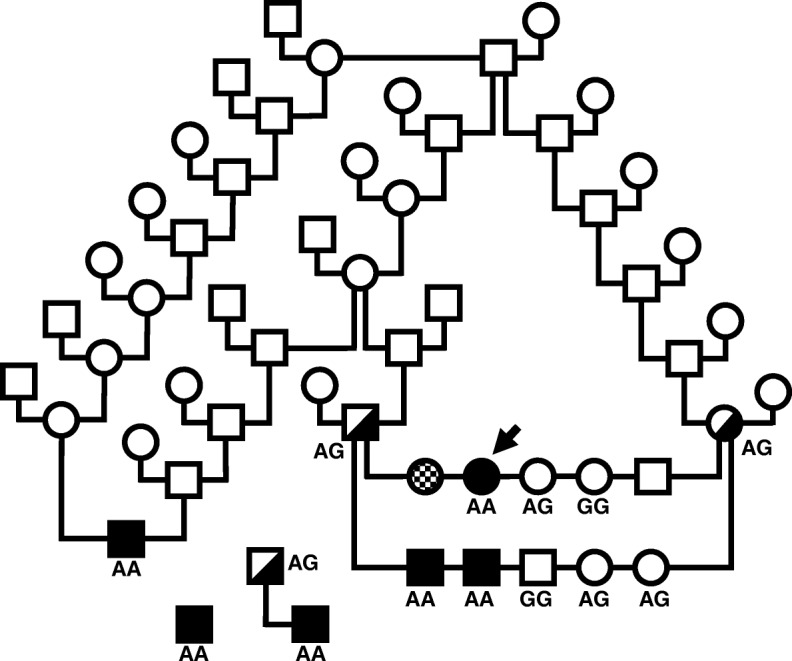
Pedigree of a Komondor dog kindred with hereditary selective intestinal cobalamin malabsorption. Squares and circles are males and females, respectively. Filled symbols indicate affected dogs. Offspring in a single litter are arranged on a horizontal line that connects lines descending from the parents’ symbols. The arrow points to the index case (case 5, Additional file 1), and the checkered symbol indicates case 4 that died without biochemical or molecular diagnosis. Half-filled symbols indicate carriers of the disease allele determine by genotyping the *CUBN* c.NM_001003148.1; c.8746 + 1G > A variant. Genotypes of dogs determined as in Fig. 3b are indicated below or immediately adjacent to their symbols. Open symbols indicate dogs which were not genotyped

Detailed clinicopathological information was available from 5 affected dogs, 3 males and 2 females that presented over a period of 12 years from both coasts of the United States and the Midwest (Additional file 1). Family history of affected Komondors, when available, indicated that parents and other littermates were clinically healthy. The 2 female affected dogs had 3 normal littermates. One of the affected females died before biochemical or molecular genetic diagnosis, but clinical signs and laboratory results indicated that it most likely suffered the same disorder as the others. The same mating subsequently produced 2 more affected males and 3 normal littermates (Fig. 2). The 2 affected dogs of the second litter were ascertained at 10 weeks of age, before the onset of clinical signs, by measuring serum cobalamin concentrations and urinary MMA excretion, and life-long parenteral cobalamin treatment prevented clinical abnormalities. The incidence of the disorder in this nuclear family (4 affected, 2 males and 2 females, of 10 total puppies) was as expected under the hypothesis of autosomal recessive inheritance (Fisher’s exact test *p* < 0.0003).

Cyanocobalamin (400–1000 μg) administered subcutaneously or intramuscularly led to rapid clinical responses. One affected dog was treated with 800 μg of cyanocobalamin spray administered to oral mucous membranes and responded well and rapidly. Cyanocobalamin administration continued with subcutaneous injections every other week to monthly or with sublingual spray daily, and the dogs remained clinically asymptomatic. Each affected puppy rapidly gained weight and became active, and all other clinical signs resolved, including tremors and seizures. The seizing dog’s phenobarbital and potassium bromide treatments were discontinued without seizure recurrence after initiating cobalamin treatment. All laboratory abnormalities resolved except for the ligand-specific mild to moderate proteinuria, recurring low serum cobalamin concentrations, and moderately elevated serum MMA concentrations.

### Molecular genetic investigations

#### Genetic exclusion analysis

Clinical, hematological, and biochemical signs compatible with cobalamin deficiency and mild proteinuria suggested dysfunction of cubam in both ileal brush border and renal proximal tubular epithelia, but there was also the possibility of IF dysfunction accompanied by proteinuria of unrelated cause [5, 23]. Cubam dysfunction is genetically heterogeneous in humans [14, 15], and dogs. In dogs, the disorder is caused by certain variants of either *CUBN* or *AMN* [16–20] that segregate within certain breeds. Variants causing IF dysfunction are confined to the *CBLIF* locus (previously designated *GIF*) and thus far have only been reported in human patients [5, 23]. To simplify interpretation of the many variants expected in an investigation by whole genome sequencing (WGS) with comparison to a single reference genome, we undertook genetic exclusion of the various candidate genes. We genotyped those members of the nuclear Komondor family segregating I-GS (Fig. 2) for which DNA samples were available, including both parents, 3 affected dogs, and 4 clinically normal littermates.

There was a fully informative SNV ~ 6 kb centromeric from the 3′ end of *AMN* and a second informative SNV ~ 2.1 kb telomeric of the 5′ end of *AMN* (Table 1; Additional file 2). Alleles of these *AMN*-flanking SNVs were in linkage disequilibrium, but none of the restriction fragment length polymorphisms (RFLPs) they created segregated with the disease locus (Table 2; Additional file 2), thus excluding *AMN* as a candidate gene. The *CBLIF* locus was similarly excluded by examining alleles of an intron 6 pentanucleotide repeat in the same nuclear family. Neither allele segregated with the disease locus (Tables 1 and 2; Additional file 2).

Because the *CUBN* gene is 259 Kb in length, we examined marker alleles near both ends of the gene (Table 1; Additional file 2). Alleles of an SSR in *CUBN* intron 4 and another in intron 56 were in linkage disequilibrium. Alleles of both SSRs were homozygous in the 3 affected dogs, heterozygous in both parents and 3 normal littermates, and homozygous wild-type in 1 normal littermate (Table 2; Additional file 2). These data failed to exclude *CUBN* from the disease locus, thus we focused our search for a disease-causing variant on that candidate gene.

#### Whole genome sequencing

Whole genome sequences of 3 I-GS affected, the sire of 1 of them, and a clinically normal Komondor (20–25 coverage each) were compared to the CanFam 3.1 canine reference genome sequence. This revealed 2 homozygous missense variants in *AMN* (NM_001002960.1) of the affected dogs: c.8C > G (p.Ala3Gly) and c.313C > T (p.Leu105Phe). Both variants were also homozygous in the clinically normal sire of 1 of the affected dogs, and both variants were considered tolerant by SIFT [24]. Similarly, there were 4 variants in *CBLIF* (NM_001005759.1) that we considered nonpathogenic because, while homozygous in an affected dog, they were also homozygous in his clinically normal sire: c.41C > T (p.Ala14Val); c.913G > A (p.Val305Ile); and c.1074-10_1074-2delAATCTTGCA (does not change the splice acceptor site of intron 7). A variant c.244A > G (p. Ser82Gly) was heterozygous in both an affected dog and his sire. All the *CBLIF* missense variants were considered tolerant by SIFT. There were no disruptive or missense variants of *TCN2*, encoding transcobalamin, in any affected dog sequenced.

In *CUBN* (NM_001003148.1) we observed 2 homozygous SIFT-tolerant variants in the affected dogs that were also homozygous in the clinically normal sire and, therefore, which we did not consider pathogenic: c.6201C > A in exon 41 (g.19,950,597; p.Phe2018Leu) and c.7175 T > C in exon 46 (g.19,960,139; p.Val2392Ala). The clinically normal Komondor’s sequence had an additional homozygous variant c.9362G > C (g.19,999,373; p.Ser3121Thr), which was tolerant by SIFT analysis.

**Fig. 3:**
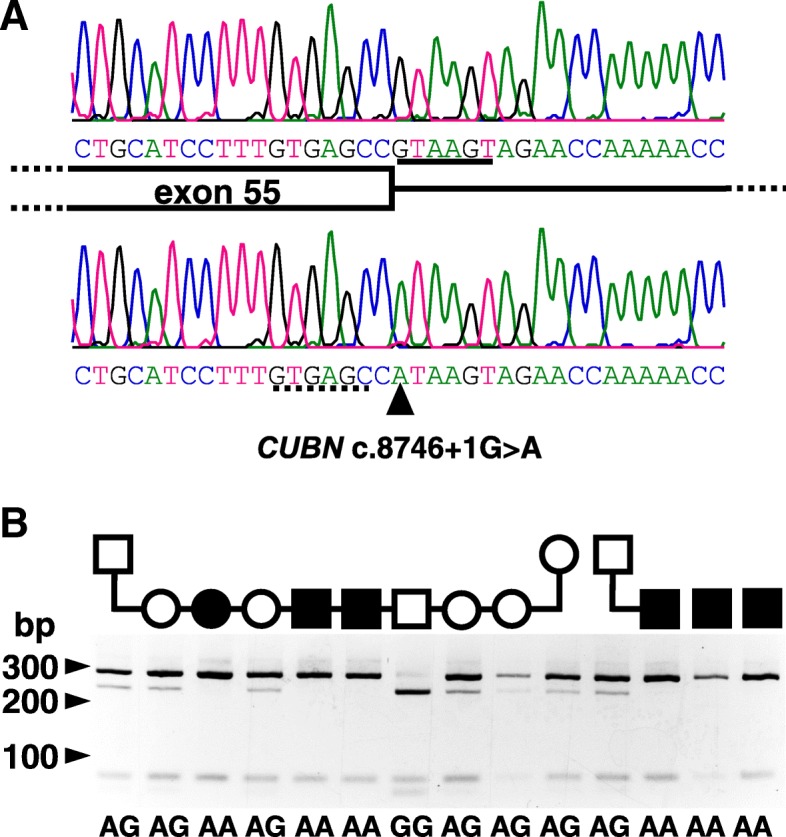
**a**. DNA sequencing electrophoretograms of the *CUBN* exon 55/intron 55 boundary. Shown are results of a normal control (above) and a Komondor dog with hereditary selective intestinal cobalamin malabsorption (below). The cartoon indicates the 3′ end of exon 55 (box) and the 5′ end of intron 55 (line) aligned with the sequences. The splice donor consensus has a solid underline in the normal dog sequence. The *CUBN* c.NM_001003148.1; c.8746 + 1G > A variant is indicated by the black triangle and a potential cryptic splice donor has a dotted underline in the affected dog sequence. **b**. Genotyping of the *CUBN* c.NM_001003148.1; c.8746 + 1G > A splicing variant in a Komondor family segregating I-GS. Genomic DNA samples were amplified by PCR using primers flanking the G/A variant of the intron 55 splice donor consensus (Table 1, Additional file 2). Bce AI digestion fragments of the PCR amplicons were separated on 4% agarose gels. An endonuclease control cut-site created 293 and 68 bp fragments from the 361 bp amplicon. Cutting at the wildtype G recognition site further cut the 293 bp fragment into 237 and 56 bp fragments. The deduced genotypes are shown below each lane, and symbols above indicate the sex and disease status of each dog as in Fig. 2

However, and of particular note, there was a *CUBN* variant (NM_001003148.1; c.8746 + 1G > A) at genomic position chr2:g.19,981,457 (CanFam 3.1), which was homozygous (A) in the affected dogs, heterozygous (A/G) in the clinically normal sire, and wildtype sequence (G/G) in the normal Komondor. This variant obliterates a splice donor consensus sequence in intron 55, predicting an abnormal RNA splicing pattern, and was thereby considered pathogenic. We confirmed the variant in all 6 affected Komondors by Sanger sequencing (Fig. 3a). A search of *CUBN* variants in the Genome Aggregation Database [25] (gnomAD; http://gnomad.broadinstitute.org, accessed Nov. 16, 2018), including WGS of 282,822 healthy humans, revealed 38 variants in the + 1 position of the 66 intronic splice donor sites (frequency of 2.0 × 10^− 6^). None were homozygous.

#### CUBN variant genotyping

We assessed segregation of the putative *CUBN* disease-allele in a family and 3 sporadic cases of Komondors with I-GS using a convenient DNA genotyping assay (Table 1; Additional file 2) of PCR to produce an amplicon flanking the variant and restriction enzyme (Bce AI) digestion to discriminate the alleles (Fig. 3b). The A allele was indicated by failure of the PCR amplicon to be digested except at the control Bce AI cut site. The 6 affected dogs were homozygous A, the 3 parental dogs were heterozygous A/G, and the 5 clinically normal littermates were homozygous G or heterozygous A/G (Table 2; Additional file 2).

We assessed Komondor I-GS allele frequencies in North America and Hungary using a TaqMan® assay. Of 98 Komondors genotyped, 12 were heterozygous, and 86 were homozygous G for the *CUBN* NM_001003148.1; c.8746 + 1G > A variant. Seven of 42 Komondors from North America (allele frequency of 8.3%) and 5 of 56 Komondors from Hungary (allele frequency of 4.5%) were heterozygous (i.e. carriers of the putative disease-allele). Pedigree analyses revealed distant relatedness among some carriers in Hungary and to some Komondors in North America, including 1 of the 3 sporadic cases (Fig. 2). The nearest common ancestors (top of Fig. 2) to all affected dogs in the United States whose pedigrees were available were dogs born in Hungary in 1968 and 1973, respectively, and subsequently imported into United States breeding programs. The *CUBN* c.NM_001003148.1; c.8746 + 1G > A variant was not observed in 100 dogs of other breeds.

## Discussion

In contrast to many dog breeds today, the Komondor is an old breed, brought originally to Hungary in the 12th and 13th centuries by the Cumans, a Turkish speaking nomadic tribe [26]. By Hungarian parliamentary decree in 2004, the Komondor is today recognized as a national treasure to be protected from genetic admixture. It is a large breed dog in which adult males average 36–45 kg and whose traditional vocation was to guard sheep and goat herds.

The early signs of hereditary selective intestinal cobalamin deficiency, including poor appetite, muscle wasting and failure to thrive, can be masked by a long hair coat or otherwise missed by owners unfamiliar with the rapid growth of healthy juvenile Komondors. More obvious signs such as seizures or collapse will bring affected dogs to veterinary attention, but their condition may be misunderstood and considered untreatable, leading to euthanasia. Failure to recognize and treat the cobalamin deficiency allows not only protracted waxing and waning of signs and progression of the disorder but potentially lethal metabolic crises complicated by hyperammonemia, ketoacidosis, and/or hypoglycemia. Megaloblastic dyshematopoiesis, neutropenia, and methylmalonic acidemia/−uria are more specific findings that support the diagnosis of this inborn error of metabolism. Serum homocysteine is also elevated in cobalamin-deficient dogs [27, 28], but it is a less specific finding than elevated MMA and is less reactive to the deficiency.

In contrast to humans, the anemia of cobalamin deficiency in dogs is not macrocytic when evaluated by Wintrobe indices [27]. The presence of some large red blood cells is masked by many misshaped and/or small RBCs, as indicated by a widened red cell distribution when evaluated by flow cytometry. Circulating megaloblasts may be observed and are too large to be misinterpreted as nucleated RBCs of other causes, such as lead poisoning. Neutropenia is a common feature of the cobalamin deficient dog hemogram, and hypersegmented neutrophils are usually evident upon careful examination of blood smears. Dyssynchrony of nuclear and cytoplasmic maturation (megaloblastosis) in the erythrocytic and myelocytic series is evident in bone marrow [27]. As shown in the affected Komondors here, age of clinical disease detection and signs varied, but intermittent parenteral cobalamin supplementation ameliorated all clinical abnormalities, except the constitutive ligand-specific proteinuria.

While cobalamin deficiency may have different causes, severe cobalamin deficiency of several related juvenile Komondors and complete recovery with parenteral cobalamin administration indicates a hereditary GI defect in this breed. Clinical observations in affected dogs showed that a parenteral megadose (400–1000 μg) of cyanocobalamin corrected the metabolic disturbances of cobalamin deficiency in affected Komondors within 2–3 days and began correction of the hemogram over the ensuing few weeks. Affected dogs recovered normal weight-for-age within 2 months. Following treatment, however, the initial supernormal serum cobalamin concentration, which may be 2–5-fold greater than the high end of the normal reference range, fell rapidly to below the normal range in about 2 weeks. This is because serum cobalamin is bound entirely to transcobalamin in dogs [29] and is rapidly translocated into cells. In contrast, 70–80% of serum cobalamin is bound to haptocorrin in humans [30] and continues to circulate in plasma. The remission of clinical signs after a single cobalamin megadose can last for 12 weeks, even in a rapidly growing juvenile, despite a gradual rise of methylmalonic acidemia above normal during that period [31]. In 1 Komondor studied as an adult, the serum methylmalonic acid concentration fell but was not fully suppressed into the normal range by a single 1000 μg cobalamin treatment, perhaps due to the large mass of the dog, and despite the dog remaining clinically normal. Thus, the rationale for deciding when to treat again cannot be based on serum cobalamin or serum methylmalonic acid determinations but ought to be rather a pre-determined interval and dosage that will keep clinical signs of deficiency from reappearing. A typical interval is every 2–4 weeks in I-GS dogs of various breeds.

Also demonstrated here in 1 of the affected Komondors (case 2), a daily dose of cobalamin (800 μg/day) administered by sublingual spray can be a successful treatment, but such a regimen does not produce a superior effect and costs more. The mechanism by which a few micrograms of an oral cobalamin megadose is absorbed in the absence of functional IF or cubam is unknown, but the phenomenon of IF-independent cobalamin absorption from the proximal small intestine exposed to large doses was observed in dogs long ago [32, 33]. The non-specific mechanism of cobalamin absorption is inefficient but explains how Minot and Murphy’s “liver therapy” could induce hematologic remission in pernicious anemia patients in the absence of IF [34]. Their recommended liver dose (120–240 g/day) contained up to 150 μg of cobalamin, or 60 times the normal daily requirement of dietary cobalamin.

We opted for genetic exclusion analyses prior to whole genome sequencing in this instance because, while the clinical disorder suggested few candidate genes, sequencing of *AMN* and *CUBN* each present difficulty. *AMN* is GC rich (65–70%) throughout the gene (GenBank® accession no. KF445236), so much so that the canine reference genome has large gaps in the *AMN* locus and is missing several exons. The *CUBN* gene is large, with 67 exons extending over 259 Kb, and again, the CanFam 3.1 reference genome is missing exon 32 (compare GenBank® accession no. AF137068 to reference genome). Of greatest concern was that without genetic positional information, the inevitably numerous sequence variants observed by WGS would be difficult to interpret. We excluded *CBLIF* and *AMN* as disease loci because alleles of internal or flanking markers did not segregate in our study kindred with alleles of an autosomal recessive disease locus.

The all-important assumption of autosomal recessive inheritance depends on the definition of the trait under study, which in this case is juvenile-onset cobalamin deficiency with compatible clinical signs and ligand-specific proteinuria. This definition does not rule out the possibility that heterozygous Komondors may have subclinically reduced cobalamin absorption. However, heterozygotes in this study were healthy, had serum cobalamin concentrations equally within the normal range as normal control dogs (data not shown), and had no detectable urinary cubam ligands (lane 2, Fig. 2). Furthermore, in a study of orally-administered radioactive cobalamin absorption in Giant Schnauzers, the heterozygous dog demonstrated cobalamin absorption equal to unrelated normal control dogs [27].

While we found several missense variants in *CUBN* by WGS, we dismissed them as causative, because they were tolerated by SIFT and were also homozygous in clinically healthy Komondor dogs. However, the intronic *CUBN* c.NM_001003148.1; c.8746 + 1G > A variant was only homozygous in affected dogs and is likely pathogenic because it disrupts the splice donor consensus sequence of intron 55. The G nucleotide in that position is 100% conserved in mammalian introns [35]. Loss of the splice donor predicts exon skipping with the loss of the 157 bp of exon 55, at a minimum, thus introducing a shift of the translation reading-frame and creating a premature stop codon that is 26 codons into exon 56. The sequence GTGAGC near the 3′ end of exon 55 (Fig. 3) might be interpreted as a cryptic splice donor but use of it would delete 7 bp from the mRNA, again causing a frame-shift and a premature stop codon at the same place in exon 56. A similar frameshift variant in I-GS affected Border Collies was demonstrated to cause ~ 10-fold reduction *CUBN* mRNA expression [17], most likely by nonsense-mediated mRNA decay [36], and failed CUBN protein expression. While expression studies are optimal for validation of variant pathogenicity, we had no access to intestinal and/or renal tissue biopsies to investigate cubam expression in these privately owned and successfully treated I-GS affected Komondors. Thus, using the American College of Medical Genetics Standards and Guidelines for interpretation of sequence variants [37], we conclude that the *CUBN* c.NM_001003148.1; c.8746 + 1G > A variant is a highly likely pathogenic null allele. The small amount of uncertainly that remains due to lack of direct experimental evidence of an effect on *CUBN* mRNA or protein expression will be further reduced as more Komondor I-GS cases come to light and are genotyped.

In mice, targeted genetic disruptions of either *AMN* or *CUBN* create embryonic lethality [38, 39]. In humans and dogs, however, many naturally-occurring null alleles of *AMN* and *CUBN*, including early (5′) frameshift variants and large deletions of the entire *CUBN* locus, have been described without evidence of embryonic or fetal developmental abnormalities [5, 14–20]. Reported missense or nonsense *CUBN* variants that cause I-GS in humans are clustered in the first 28 exons, which encode regions important for IF-cobalamin binding and AMN-mediated membrane localization and internalization (Human Gene Mutation Database, accessed July 27, 2018; http://www.hgmd.cf.ac.uk/ac/index.php), [5]. In contrast, the 3 known I-GS-causing *CUBN* variants of dogs are 2 single-base deletions (c.786delC in Beagles and c.8392delC in Border Collies), and the splice-site variant reported here, each predicting a frameshift [17–20]. Two of these are very distal in the gene, well 3′ of exons encoding the IF-cobalamin-binding site in CUB domains 5–8, and emphasize the importance of mRNA stability in gene expression.

The ascertainment of human I-GS patients suggests that cobalamin malabsorption is the single aspect of cubam dysfunction that alerts medical clinicians to the need for treatment intervention and molecular investigation. Urinary loss of the cubam ligands, albumin and vitamin D-binding protein, may cause phenomena such as low-grade tubular albuminuria [8, 40] or a subclinical reduction of hydroyxlated forms of vitamin D in serum. A study in cobalamin treated, affected dogs of the I-GS Giant Schnauzer line demonstrated significantly increased 25-hydroxyvitamin D_3_ concentrations in urine, due to failed tubular reabsorption of vitamin D binding protein, and ~ 55% decreases of serum 25-hydroxyvitamin D_3_ and 1,25-dihydroxyvitamin D_3_ concentrations in serum [6]. Despite these biochemical abnormalities, we and others have observed no consequences of the proteinuria to the health of adequately cobalamin-treated I-GS affected dogs, regardless of breed and, therefore, gene or sequence variant [27, 28, 31, 41–43].

The *CUBN* c.NM_001003148.1; c.8746 + 1G > A variant permitted design of simple RFLP and Taqman® assays to differentiate between homozygous affected, asymptomatic heterozygous, and wildtype healthy Komondors. While we did not pursue a randomized survey, screening of Komondors in North America revealed an 8.3% prevalence of the disease-allele in this small population. Moreover, surveying Komondors in Hungary, the breed’s country of origin, revealed a disease-allele prevalence of 4.5%. Common ancestors trace back to dogs born in Hungary in the early 1970s. Thus, the I-GS allele has been segregating in Komondors for decades and is widespread in the breed worldwide. The higher allele-frequency in North America could be due to a founder effect subsequent to importation of a popular sire or dam.

## Conclusions

Breed-specific cubam mutations that cause life-threatening cobalamin deficiency in dogs now include a *CUBN* splice-site variant (*CUBN* c.NM_001003148.1; c.8746 + 1G > A) segregating among Komondors. The disease-allele appears to be widespread among Komondors in North America and Hungary. Simple and reliable allele-specific DNA tests are available so that Komondors with clinical signs suggestive of I-GS may be diagnosed early and treated effectively. We also recommend that Komondors intended for breeding be tested to identify carriers and prevent production of additional affected dogs.

## Methods

### Animals, clinicopathologic and metabolic studies

These investigations included clinical case studies and/or laboratory analyses of samples from 4 I-GS affected Komondors, their parents, and 6 healthy littermates in 1 nuclear family plus 3 affected Komondors that occurred as sporadic cases and 1 of their sires. Owners submitted samples for DNA isolation and genotyping from 98 additional clinically-normal Komondors in North America and Hungary to assist with their breeding programs. Additionally, we studied DNA samples from 100 dogs of other breeds that were in laboratory archives. Blood and urine sampling from Komondors were according to principles and protocols approved by the respective Institutional Animal Use and Care Committees at Michigan State University, University of Pennsylvania, and University of Tennessee. Owners and local veterinarians submitted case histories and samples for routine diagnostic tests such as blood tests, DNA isolation, metabolic analysis, and urinalysis from dogs of Komondor kindreds or sporadic cases. Pedigrees were gathered to determine shared ancestry. Characterization of cobalamin-deficient dogs included clinical signs, medical history, routine complete blood counts, serum chemistry panels, serum cobalamin and folate concentrations, examination of bone marrow aspirates, and routine and special urinalyses.

In affected dogs, excess methylmalonic acid excretion in urine was documented either qualitatively by a specific reaction with diazotized p-nitroaniline or quantitatively by gas chromatography-mass spectroscopy. Urine proteins were concentrated by centrifugation through a 10 kDa molecular weight cut-off membrane and separated by one-dimensional SDS-polyacrylamide gel electrophoresis, and protein identities were confirmed by antibody reactivity on immunoblots, as previously described [6–9, 17, 18].

### Molecular genetic investigations

#### Genetic exclusion analysis

Genomic DNA was prepared from buccal brushes or EDTA-anticoagulated whole blood by standard methods [16]. Single nucleotide variants (SNVs) and polymorphic simple sequence repeat markers (SSRs) in or closely flanking the genes *CBLIF, CUBN* and *AMN* were chosen from a canine reference genome sequence (Broad/CanFam 2.1, May 2005) as viewed on the University of California Santa Cruz genome browser [44] with RepeatMasker [45] and Single Nucleotide Polymorphism database (dbSNP build 131) tracks [46]. However, coordinates given in this report (Table 1; Additional file 2) are updated to the Broad/CanFam 3.1 assembly, September 2011. For polymerase chain reactions (PCR) we used standard primer, deoxynucleotide, and genomic template concentrations, 1.5 mM MgCl_2_, and 2 U *Taq* polymerase (Invitrogen Corp., Carlsbad, CA) in 50 μL reactions. For higher GC templates 1 M betaine was added to the reaction, as noted in Table 1; Additional file 2. Marker genotyping was by restriction enzyme digestion and/or Sanger sequencing of PCR amplicons. Sequences and accompanying electrophoretograms were viewed in the SeqMan program of the Lasergene sequence analysis suite (DNASTAR®, Inc., Madison, WI).

#### Whole genome sequencing (WGS)

DNA samples of 5 Komondor dogs, including 3 affected dogs of widely separated ancestry, 1 clinically normal sire, and 1 clinically normal littermate of an affected dog were prepared for WGS. Genome libraries were prepared using Illumina® TruSeq Nano LT Kit (FC-121-4001 & FC-121-4002; Illumina®, San Diego, CA). Briefly, genomic DNA was fragmented, end-repaired and adenylated before ligation with the corresponding unique index adapters and amplified for WGS. All samples were sequenced on the Illumina HiSeq 2500 platform by paired-end sequencing. Average genome sequence coverage for each dog was 20-25X (The datasets generated and analyzed during the current study are available in the BioProject ID: PRJNA486818 repository, http://www.ncbi.nlm.nih.gov/bioproject/486818 and BioSample accessions: SAMN09865705, SAMN09865706, SAMN09865707, SAMN09865708, SAMN09865709; Release date: 2019-01-01.

Genomic data were searched for variants using the CanFam 3.1 reference sequence with a focus on the potential candidate genes *CUBN*, *AMN*, *CBLIF*, and *TCN2*. Disruptive variants, homozygous in the affected dogs and heterozygous in the parental dog were prioritized as potential disease-causing candidates. Functional effects of missense variants were examined by Sorting Intolerant From Tolerant (SIFT) analysis [24] and resequencing DNA of clinically affected and normal dogs.

#### CUBN variant genotyping

The sequence variant *CUBN* c.NM_001003148.1; c.8746 + 1G > A (chr2:g.19,981,457 CanFam 3.1) was genotyped in all members of the I-GS Komondor kindred and sporadic cases using PCR amplification primers as listed in Table 1; Additional file 2, followed by Bce AI restriction digestion of the amplicon (New England BioLabs®, Inc., Ipswich, MA). Digested amplification products were separated on 4% agarose gels. An allelic discrimination real time PCR assay was designed for population-wide genotyping of the NM_001003148.1; c.8746 + 1G > A variant (Forward Primer: CAGGGCTTCTCTGCATCCT; Reverse Primer: GACCTCCCGGGTTGGTTTT; Probe 1 (VIC Dye): TTGTGAGCCGTAAGTAG; Probe 2 (FAM Dye): TTGTGAGCCATAAGTAG). Primers and probes were obtained as a Custom Taqman® SNP Genotyping Assay (Applied Biosystems, Life Technologies, Grand Island, NY). A total of 98 Komondors from North America and Hungary and archived samples from 100 other dogs of different breeds were screened using the TaqMan® assay following standard conditions.

## Additional files

Additional file 1 Format is Adobe Portable Document Format (.pdf), titled Detailed clinical descriptions and course of disease before and following specific treatment. The file summarizes 4 clinical cases of I-GS in young Komondors as they were presented to veterinary clinicians across the USA. (DOCX 22 kb)

Additional file 2 Format is Adobe Portable Document Format (.pdf), titled Genetic exclusion markers. The file contains 2 tables: Table 1 describes genetic exclusion markers used to query biological candidate genes for segregation of alleles with the disease locus, and Table 2 shows alleles of the markers described in Table 1 in each member of a family of Komondors segregating I-GS. (DOCX 33 kb)

## Abbreviations

*AMN*: Amnionless
*CBLIF*: Cobalamin binding intrinsic factor
cubam: the functional receptor complex composed of AMN and CUBN protein subunits
*CUBN*: Cubilin
dbSNP: Single nucleotide polymorphism database
EDTA: Ethylenediaminetetraacetic acid
IF: Intrinsic factor
I-GS: Imerslund-Gräsbeck syndrome
MMA: Methylmalonic acid
PCR: Polymerase chain reaction
RFLP: Restriction fragment length polymorphism
SIFT: Sorting intolerant from tolerant
SNV: Single nucleotide variant
SSR: Polymorphic simple sequence repeat markers
SW: Smith-Waterson
WGS: Whole genome sequencing; the three-letter amino acid code is used throughout.
