# Supplementary material for: Inherited selective cobalamin malabsorption in Komondor dogs associated with a CUBN splice site variant
Source: BMC Vet Res. 2018 Dec 27;14:418. doi: 10.1186/s12917-018-1752-1 (PMC6309081; doi:10.1186/s12917-018-1752-1)
Supplement: Supplementary file 1 — Format is Adobe Portable Document Format (.pdf), titled Detailed clinical descriptions and course of disease before and following specific treatment. The file summarizes 4 clinical cases of I-GS in young Komondors as they were presented to veterinary clinicians across the USA. (DOCX 22 kb) [file 12917_2018_1752_MOESM1_ESM.docx]

**Additional File 1:** **Detailed clinical descriptions and course of disease before and following specific treatment.**

**Case 1**

A six-month-old, male, neutered Komondor presented to a primary care veterinarian with a three-month history of progressive inappetence, weakness, diarrhea, and weight loss. He was up to date on vaccinations and had been dewormed. On the day of presentation, he had been inappetent and lethargic, with intermittent vomiting and a fine head tremor. He collapsed upon standing and was brought to the clinic for evaluation. At presentation, the puppy was very underweight at 13.3 kg (BCS 1/9). Vital parameters and neurologic examination were within normal limits.

Complete blood count revealed non-regenerative, mildly microcytic anemia (Hct 20.7%), The WBC concentration was 8.7x10^3^/μL (N 4.9-17.6 10^3^/μL), and the blood chemistry results showed neutropenia (1,244 cells/μL), monocytopenia (162 cells/μL), and thrombocytopenia. Serum biochemistry revealed mild azotemia (BUN 63 mg/dL) but normal serum creatinine concentration suggesting intestinal bleeding, a high anion gap indicating a metabolic acidosis, and mild hyperphosphatemia. Blood smear review revealed a moderate leukopenia, and many of the neutrophils appeared moderately toxic. Several hypersegmented neutrophils were also present. Urine pH and specific gravity were 6.5 and 1.043, respectively. Moderate proteinuria (+2/+3) was detected. Urine metabolic screen revealed highly elevated methylmalonic acid concentration (37,337 mmol/mol creatinine, normal <2 mmol/mol creatinine). Serum bile acids were within normal limits, ruling out liver disease or a portosystemic shunt. Abdominal and thoracic radiographs were unremarkable. Serum cobalamin levels were below the level of detection (<200 ng/L; normal 200-800 ng/L).

The dog was prescribed subcutaneous cobalamin injections at a dose of 1000 µg/week, completely recovered and was maintained on a 2-3 week interval for injections.

**Case 2**

A five-month-old, male, intact Komondor presented for chronic failure to thrive, cachexia, and inappetence to a veterinarian. The dog had experienced intermittent episodes of vomiting and diarrhea. The puppy was up to date on vaccinations, and tested negative for heartworm*, Ehrlichia canis/ewingii, Borellia burgdorferi, and Anaplasma phagocytophilum* and *A. platys*. He was being treated for an intestinal endoparasitic infection with praziquantel/pyrantel pamoate.

Complete blood count revealed a hematocrit of 38.6% and a WBC count of 7.2x10^3^/μL (N 4.9-17.6 10^3^/μL), which was considered low for a puppy his age. Neutrophils and lymphocytes were reported at 3,686/μL (N 2,940-12,670/μL) and 2,621/μL (N 1,060-4,950/μL), respectively. Moderate proteinuria was detected (2+). Urine pH was 5.5 and the specific gravity was 1.040. Serum biochemistry revealed mild azotemia (BUN 37.5 mg/dL) and mild hyperphosphatemia. Serum cobalamin levels were persistently <150 ng/L (N 251-908 ng/L).

At 7-months of age, the dog remained in poor body condition, weighing 17.2 kg. At that time, he was prescribed 800 µg/day sublingual cyanocobalamin spray. His lethargy and inappetence were reversed within 3 hours of receiving the cyanocobalamin spray. Continuing with this daily dosage of cyanocobalamin, he gained weight – weighing approximately 45 kg as an adult. As dog showed intermittently some signs of lethargy and persistently had a low serum cobalamin concentration the dog was switched to subcutaneous injections every three weeks - and has shown no further signs of illness.

**Case 3**

A 13-week-old, male Komondor was received by an owner from the breeder in seemingly good health and up-to-date on vaccinations. Upon first examination, the puppy appeared to be within normal limits, but was noted to be thin at 7.0 kg (BCS 4/9). At 14-weeks he was presented with complaints of intermittent vomiting, inappetence, and lethargy, and failure to gain weight (6.5 kg). Physical examination revealed the puppy to be depressed, lethargic, thin, vomiting, and dehydrated. Complete blood count revealed mild anemia (Hct 23.4%) and severe neutropenia (900/μL). He was hospitalized for 2 days and received IV physiological NaCl, ampicillin, metronidazole, and metoclopramide.

At 15-weeks, the puppy was presented to an emergency clinic for a seizure. He experienced 2 additional seizures during the night, which were controlled with IV valium. Mild anemia, lymphopenia, elevated anion gap (18.1), and mild azotemia were also documented. His weight remained at 6.5 kg (normal male Komondor weight at this age being ~22 kg). Neurological examination revealed that the dog was mentally dull, stuporous, and tetraparetic. Pre- and post-prandial bile acids were normal. Complete blood count showed mild lymphopenia. Urinalysis revealed moderate protein (2+), and the urinary MMA spot test was positive.

The puppy was started on oral phenobarbital (28 mg twice daily), intravenous fluids, and oral thiamine (50 mg). His treatment was changed to levetiracetem (Keppra) (20 mg/kg TID), and cyanocobalamin (250 µg SQ SID). Pretreatment serum cobalamin was <150 ng/L, and urine MMA was 20,320 mmol/mol creatinine. Cubam ligands were identified in the urine. The dog was released on Keppra and 400 µg cyanocobalamin once daily for 7 days, then every 30 days.

At recheck, 6-weeks following initiation of cyanocobalamin treatment, the dog was seizure-free, weighing 16.1 kg. Clinical and neurologic examinations were normal. Serum cobalamin concentration was 231 ng/L (N 251-908 ng/L). The cyanocobalamin dose was increased to 600 µg weekly because the dog was gaining weight. At 11-months of age the dog remained seizure-free on potassium bromide. He weighed 50 kg and was reported as alert, active, and growing.

The dog was presented to a veterinarian at 3 years, 7 months of age with immune-mediated thrombocytopenia. He had IgG and IgM anti-platelet antibodies in plasma but no anti-C3 antibodies. Complete blood count showed reticulocytosis (204,000/µL), low platelets (6,000/µL) and high platelet volume (21.9 fL). He was treated with prednisone, azathioprine, and vincristine. Serum cobalamin measured 865 ng/L shortly after treatment, but the serum MMA concentration remained elevated at 3,188 nM (N 415-1,193 nM). Cyanocobalamin dosage was increased to 2000 µg/month.

**Case 4:**

An 8-month old, female intact Komondor puppy presented to a referral hospital, with a 3-month history of decreased appetite, lethargy, reluctance to move and cyclic fevers that occurred monthly. Diagnostic tests done prior to presentation included a complete blood count, serum chemistry profile and total T4 concentration, and abdominal radiographs. The CBC was unremarkable, and the total T4 level was within normal limits. The serum chemistry profile was consistent with that of a young growing dog. The abdominal radiographs showed no remarkable abnormalities. Treatment prior to presentation included long term oral broad-spectrum antibiotics, 1 injection of corticosteroids, and carprofen to be given at home. When there was no response to therapy an abdominal exploratory surgery was then performed. The only reported abnormality was a hyperemic pancreas. No biopsies were obtained.

Physical exam at the referral clinic revealed a temperature of 102.3° F, pulse was 120 bpm, and respiratory rate was 40 rpm. The current weight was 13.3 kg (normal for age 30-40 kg). The dog had thin body condition with poor muscle mass. She exhibited pain on manipulation of the head and neck region and on palpation of the cervical and thoracic spine. A neurologic examination was performed with the only finding being pain. The main problems identified included poor growth and thin body condition, decreased appetite, cyclic fevers, neck pain, and a non-regenerative anemia.

There was moderate non-regenerative anemia with a hematocrit of 27.7% (37-55) wh itslight anisocytosis and poikilocytosis The cerberospinal fluid showed neutrophilic to mixed cell leukocytosis consistent with inflammation and negative bacterial culture.

The dog was discharged with prednisone at a dose of 2 mg/kg/day and famotidine 0.5 mg/kg/day. The dog was re-checked 2 weeks later and was reported to be doing better with an increased appetite, activity, weight gain and a resolution of the pain and fevers. On re-check examination temperature, pulse, and respiration were within normal limits and the body weight was 15 kg. Three weeks after starting immunosuppressive therapy the dog was lethargic, anorexic, and vomiting. Upon presentation to the referral clinic at 10 months of age, the dog was recumbent and poorly responsive. The current weight was 16 kg, temperature 101.2^o^F, respiratory rate was 40 bpm and heart was pounding with a rate of 160 bpm. No neck pain was evident. Packed cell volume was 12%. Crystalloids and a unit of canine packed red blood cells were administered. A bone marrow aspirate was done under light sedation. The bone marrow aspirate findings were consistent with a non-regenerative anemia with some evidence of erythroid line dysplasia and megaloblastic ineffective erythropoiesis. The blood and bone marrow findings fit the criteria for mild myelodysplastic syndrome. The dog responded poorly to fluid therapy, broad spectrum antibiotics and whole blood transfusion. The dog became febrile (104^o^F) and died the same evening.

**Case 5**:

A littermate to case 4, owned by the same family, was presented with similar lethargy and anemia 2 weeks later. A third littermate had been recently acquired to replace the dog of case 4, and it was noted to be much heavier and more muscled than the first 2 juvenile dogs. Case 5 weighed 11.7 kg and the clinically normal littermate weighed 29.4 kg. The complete blood cell count of Case 5 showed a leukopenia (4.3 x10^3^/uL [5.7-16.3]) and anemia (hematocrit of 27.7% [35-55]) with mild regeneration (absolute reticulocyte count 64,980 [0-60,000]). Urinalysis showed specific gravity 1.013, pH 5.5, and trace proteins. An ACTH stimulation test was within normal limits, but total T4 was low at 0.4 µg/dL (1-4). The serum cobalamin concentration was <150 ng/L (normal: 284-836 ng/L), and serum folate was normal. Urine methylmalonic acid concentration was increased at 8,900 mmol /mol creatinine (< 2 mmol/mol creatinine) and 28 mmol methyl citrate /mol creatinine. Methyl citrate is an abnormal metabolite made by an alternative pathway when MMA-CoA cannot be processed through the usual cobalamin-dependent pathway and is typically undetectable in urine.

The dog was treated with 500 µg cyanocobalamin intramuscularly. Within 1 week of treatment the hematologic abnormalities had resolved, and the dog had gained 5 kg . Treatment continued with 500 μg of cyanocobalamin given weekly for 4 weeks followed by 500 μg of cyanocobalamin given monthly indefinitely.

It was discovered that the same parents were bred once again producing a litter of 5 puppies. At 10 weeks of age the new puppies were screened for the disease with serum cobalamin and urine MMA determinations. Two male puppies were found to be affected and accordingly treated.

| **Table 1:** Urinalysis results from 4 Komondors with confirmed I-GS. | | | | | | | | | |  |
| --- | --- | --- | --- | --- | --- | --- | --- | --- | --- | --- |
| **Case** | **pH** | | **Specific Gravity** | **Proteinuria**  **0 to 4+** | | | **MMA Concentration** | | |  |
|  |  |  |  |  |  |  | **Spot Test**  **(0 to 4+)** | **mmol/mol creatinine** | |  |
| 1 | 6.5 | | 1.043 | +2 | | | 4+ | 37,337 | |  |
| 2 | 5.5 | | 1.040 | +2 | | | 4+ | Not done | |  |
| 3 | Not Done | | 1.040 | +2 | | | 4+ | >20,320 | |  |
| 5 | 5.5 | | 1.013 | trace | | | 4+ | 8,900 | |  |
| **Table 2:** Blood chemistry values from four Komondors with confirmed I-GS. | | | | | | | | | | |
| **Case** | | **Hematocrit** | | | **WBC** | **Neutrophil** | | | **BUN/Creatinine Ratio** | |
| 1 | | 20.7% | | | 8.7x10^3^ /μL | 1,244 /μL | | | 126 | |
| 2 | | 38.6% | | | 7.2x10^3^ /μL | 3,686/μL | | | 37.5 | |
| 3 | | 23.4% | | | 2.7x10^3^ /μL | 900/μL | | | 68.3 | |
| 5 | | 24% | | | 4.1x10^3^/ μL | 2,000/ μL | | | 25.5 | |

| **Table 3:** Summary of clinical signs in 4 Komondors with confirmed I-GS. | | | | | | |
| --- | --- | --- | --- | --- | --- | --- |
| **Case** | **Clinical Signs** | | | | | |
|  | **Inappetence** | **Failure to Thrive** | **Diarrhea** | **Vomiting** | **Head Tremor** | **Seizures** |
| 1 | ✓ | ✓ | ✓ | ✓ | ✓ | - |
| 2 | ✓ | ✓ | ✓ | ✓ | - | - |
| 3 | ✓ | ✓ | ✓ | ✓ | - | ✓ |
| 5 | ✓ | ✓ | ✓ | ✓ |  |  |
