# Supplementary material for: Inherited selective cobalamin malabsorption in Komondor dogs associated with a CUBN splice site variant
Source: BMC Vet Res. 2018 Dec 27;14:418. doi: 10.1186/s12917-018-1752-1 (PMC6309081; doi:10.1186/s12917-018-1752-1)
Supplement: Supplementary file 2 — Format is Adobe Portable Document Format (.pdf), titled Genetic exclusion markers. The file contains 2 tables: Table 1 describes genetic exclusion markers used to query biological candidate genes for segregation of alleles with the disease locus, and Table 2 shows alleles of the markers described in Table 1 in each member of a family of Komondors segregating I-GS. (DOCX 33 kb) [file 12917_2018_1752_MOESM2_ESM.docx]

| **Marker and position in CanFam 3.1 assembley** | **Site** | **PCR primers 5’ -> 3’** | **PCR assay conditions** | **Restriction enzyme** | **Alleles** |
| --- | --- | --- | --- | --- | --- |
| rs24558868 (chr8:g.70,813,790) | 3’ of *AMN** | gcgcaaagatctgtggttgc  ccctcgtctcagcctggaat | 1M betaine;  52° C anneal | BssS I | G/A |
| rs24499721  (chr8:g.70,798,303) | 5’ of *AMN* | acctgcccactcggaggaa  ctggtctcggtgacccggta | 1M betaine;  52° C anneal | Xma I | C/T |
| *CBLIF*** SSR  chr21:g.50,340,309TTTTG[4],[5]  SW score 216 | *CBLIF*  intron 6 | tgccaggagcaccgaggcttta  acctggccgatgcaccaaacat | 64° C anneal | Sanger  sequence | 4 / 5  TTTTG repeats |
| *CUBN**** SSR  chr2:g.19,786,945GAAG[13],[14]  SW score 828 | *CUBN* intron 4 | attctttcccttgtcctctgc  aaactgctcagatttcattacca | 58° C anneal | Sanger  sequence | 13 / 14  GAAG repeats |
| *CUBN* SSR  chr2:g.19,988,734TAGA[9],[11]  SW score 605 | *CUBN* intron 56 | cctggaggatctggctttacacctca  ccttggactagagcccatgccata | 64° C anneal | Sanger  sequence | 9 / 11  TAGA repeats |
| *CUBN* NM_001003148.1;  c.8746+1G>A  chr2:g.19,981,457 | *CUBN* intron 55 | ggaagtcgccgagtccctgtta  ggccttctgtgtcatctctccat | 64° C anneal | Bce AI | G/A |

**Genetic Exclusion Markers**

**Table 1.** **Genotyping markers, PCR primers and restriction enzymes.**

* CanFam 3.1 genomic (appoximate) position of *AMN* is chr8:g.70,800,372-70,807,893.

** CanFam 3.1 genomic position of *CBLIF* is chr21:g.50,334,769-50,350,598.

*** CanFam 3.1 genomic position of *CUBN* is chr2:g.19,784,125-20,041,840.

**Marker**

| **3’ *AMN* SNP** | **G/A** | **G/G** | **G/G** | **G/G** | **G/A** | **G/A** | **A/A** | **A/A** | **G/G** | **G/A** |
| --- | --- | --- | --- | --- | --- | --- | --- | --- | --- | --- |
| **5’ *AMN* SNP** | **C/T** | **C/C** | **C/C** | **C/C** | **C/T** | **C/T** | **T/T** | **T/T** | **C/C** | **C/T** |
| ***CBLIF* SSR** | **4/5** | **ne*** | **4/4** | **ne** | **4/4** | **4/5** | **4/4** | **4/5** | **ne** | **4/5** |
| ***CUBN* intron 4 SSR** | **13/14** | **ne** | **14/14** | **13/14** | **14/14** | **14/14** | **13/13** | **13/14** | **13/14** | **13/14** |
| ***CUBN* intron 56 SSR** | **9/11** | **9/11** | **9/9** | **9/11** | **9/9** | **9/9** | **11/11** | **9/11** | **9/11** | **9/11** |
| ***CUBN* c.8746+1G>A chr2:g.19,981,457** | **A/G** | **A/G** | **A/A** | **A/G** | **A/A** | **A/A** | **G/G** | **A/G** | **A/G** | **A/G** |

**Table 2.** **Marker alleles in a Komondor family segregating I-GS.**

*ne indicates samples not examined.
